# Use of mental health treatment plans, psychological treatment services and antidepressants in young Australian women: A cohort study

**DOI:** 10.1177/00048674251362038

**Published:** 2025-08-25

**Authors:** Louise F Wilson, Annette J Dobson, Katharine A Wallis, Jenny A Doust, Gita D Mishra

**Affiliations:** 1 Australian Women and Girls’ Health Research (AWaGHR) Centre, School of Public Health, The University of Queensland, Brisbane, QLD, Australia; 2 General Practice Clinical Unit, Medical School, The University of Queensland, Brisbane, QLD, Australia

**Keywords:** Mental health, women, social factors, primary care, health service research

## Abstract

**Background:**

Australia has a high mental illness burden, especially among young women. It is known that people in urban areas, with more education and higher incomes are more likely to use Better Access services (mental health treatment plans and psychological treatments), while those in rural areas, or with lower education or incomes, disproportionately use antidepressants. During the COVID-19 period, the Australian government increased access to mental health care. Our aim was to investigate how rurality, education level and perceived ability to manage with income influenced young women’s use of mental health treatment plans, psychological treatments and antidepressants separately or in various combinations (2019–2022).

**Methods:**

Survey and linked administrative data from 7642 women from the Australian Longitudinal Study on Women’s Health were used. Relative risk ratios and 95% confidence intervals for associations between sociodemographic factors and use of mental health treatment plans and treatments were estimated using multinomial logistic regression.

**Results:**

Women in rural/remote areas (vs metropolitan areas) were less likely to have a mental health treatment plan (with/without antidepressants), difficulty managing with available income (vs not too bad/easy) was associated with having a mental health treatment plan and using antidepressants. High school-educated women (vs university-educated) were more likely to use antidepressants only (relative risk ratio = 1.60; 95% confidence interval = [1.24, 2.07]). Among women with mental health treatment plans (*n* = 3525), those in rural/remote areas (relative risk ratio = 2.00; 95% confidence interval = [1.13, 3.53]) and women not university-educated were more likely to use antidepressants without psychological treatment.

**Conclusion:**

Sociodemographically disadvantaged young women disproportionately used antidepressants without Better Access services. Evidence-based interventions to reduce these inequities should be a priority.

## Introduction

Estimates suggest that the lifetime prevalence for any mental disorder is approximately 30%, with age at onset highest from childhood to young adulthood (McGrath et al., 2023). Australia has one of the highest levels of disease burden due to mental disorders worldwide (GBD 2019 Mental Disorders Collaborators, 2022), with the greatest burden experienced by women aged between 15 and 44 years (Australian Institute of Health and Welfare, 2023a).

Primary care is one of the main gateways for patients to access treatments for mental health disorders. The General Practice Health of the Nation 2023 Report highlighted mental health issues as the most common reason for a general practitioner (GP) visit in Australia (The Royal Australian College of General Practitioners, 2023). Australia has a universal health insurance scheme that covers a wide range of medicines, health and hospital services at low or no cost to all Australian citizens and permanent residents. In 2021–2022, approximately 11% of the Australian population received a mental health service (including services provided by psychiatrists, clinical psychologists, GPs and other allied health services) subsidised under the Medicare Benefits Schedule (MBS) (Steering Committee for the Review of Government Service Provision, 2024); while an estimated 17% of women and 10% of men (aged 10 years and older) were using antidepressants subsidised under the Pharmaceutical Benefits Scheme (PBS) in 2019 (De Oliveira Costa et al., 2023).

A key service delivered in primary care is the Better Access Initiative that provides subsidised services to ‘patients with a diagnosed mental health disorder who would benefit from a structured approach to the management of their treatment’. If a GP determines that a patient may benefit from psychological treatment, they can prepare a mental health treatment plan (MHTP) which allows the patient to receive subsidised services, most commonly from a psychologist, with a capped number of services available per calendar year.

The psychological treatment services available under the Better Access Initiative are most likely to be used by people diagnosed with depression/anxiety or with the greatest psychological distress (ascertained using standardised self-report measures of mental health) (Byles et al., 2011; Dolja-Gore et al., 2018; Pirkis et al., 2022). However, there is evidence of geographic and socioeconomic inequities, with people in urban areas, those on higher incomes, and with more education making disproportionate use of these services (Byles et al., 2011; Dolja-Gore et al., 2018; Meadows et al., 2015; Pirkis et al., 2022). In contrast, although not recommended as the first line of treatment for mood disorders (Malhi et al., 2021), antidepressant use (as an adjunct, or an alternative, to other treatment services) is more common in regional areas and among people who are socioeconomically disadvantaged (Australian Commission on Safety and Quality in Health Care and the National Health Performance Authority, 2015; Halme et al., 2023). Furthermore, women are 1.5 times more likely than men to be prescribed antidepressants (Wallis et al., 2024).

Out-of-pocket costs for mental health services have risen over the past decade, and the proportion of services where an out-of-pocket cost is incurred has also risen, increasing the financial barrier to accessing subsidised mental health services (Rosenberg et al., 2022). In 2021, 65% of psychological treatment services incurred a cost to the consumer (Pirkis et al., 2022). Other significant barriers to mental health services use include the inequitable distribution of the mental health workforce (especially clinical psychologists) (Meadows et al., 2015), health literacy levels and fear of stigma (Cronin et al., 2025).

During the COVID-19 pandemic, the prevalence of mental health issues increased, due to factors such as anxiety about the disease, lockdown restrictions and impacts on employment (Aknin et al., 2022; Fisher et al., 2020). In response, the Australian government widened subsidised access to telehealth and phone consultations provided by GPs and psychologists (from March 2020) and increased the annual number of subsidised psychological treatment services from 10 to 20 treatments (October 2020 to December 2022) (Australian Institute of Health and Welfare, 2022).

Previous studies on the use of Better Access Initiative services have used area-level rather than individual-level characteristics (Meadows et al., 2015), looked only at the use of psychological treatments (Byles et al., 2011; Dolja-Gore et al., 2018) or MHTPs and psychological treatments combined (Pirkis et al., 2022). Another study has examined income inequities in the probability of receiving psychological treatment and/or medication; however, this was a study only of adolescents who had an MHTP (Black et al., 2024). It remains unclear how in an adult population, sociodemographic factors are associated with use of MHTP, psychological treatment and antidepressants separately or in combination.

Therefore, the aim of our study was to investigate how remoteness of residence, education level and perceived ability to manage with available income in a study population of young Australian women influenced the separate and joint use of Better Access services and antidepressants between 2019 and 2022, spanning the COVID-19 period when the government was increasing access to mental health care.

## Methods

### Study population

The Australian Longitudinal Study on Women’s Health (ALSWH) is a prospective population-based study designed to explore factors that influence women’s health and well-being and their use of health services across different life stages. The study began in 1996, with the recruitment of three cohorts of women (born in 1973–1978, 1945–1951 and 1921–1926) (Dobson et al., 2015). In 2012–2013, a cohort of younger women born in 1989–1995 were recruited through a range of conventional and online methods (Loxton et al., 2018). This study used data for this youngest cohort from the survey conducted in 2019, when women were aged 24–29 years.

### Exposures: sociodemographic factors

Our sociodemographic exposure variables were remoteness of residence [using the Modified Monash Model, a geographic classification to objectively describe geographical access to services (Versace et al., 2021)], highest educational qualification level and perceived ability to manage with available income.

### Covariates

Levels of psychological distress, annual number of GP visits and private health insurance were included as covariates as they were identified from the literature as factors associated with use of MHTPs/ psychological treatnent services and/or antidepressants (Australian Institute of Health and Welfare, 2023b; Dolja-Gore et al., 2018; Halme et al., 2023; Meadows et al., 2015; Pirkis et al., 2022). Age was not included as a covariate because of the narrow 5-year age range of participants. State/territory of residence was not included due to collinearity between this variable and remoteness of residence.

#### Psychological distress measures

We used the Kessler Psychological Distress Scale-10 (K10) as the measure of psychological distress in our analysis. The K10 is a 10-item screening scale of non-specific psychological distress designed for use in population health surveys (Kessler et al., 2003). It has been assessed as a screening tool to identify likely cases of anxiety and affective disorders in community settings (Andrews and Slade, 2001). A single measure of the K10 has been shown to be a useful proxy for longer-term chronic psychological distress, with the experience of symptoms of distress broadly stable over up to 8 years of follow-up (Welsh et al., 2020). Total scores range from 10 to 50. Higher scores indicate a greater likelihood of psychological distress. We categorised the K10 scores using cut-points that have been used in primary healthcare and specialist treatment settings: ‘10–19 indicating no psychological distress’, ‘20–24 for mild distress’, ‘25–29 for moderate distress’ and ‘30–50 for severe distress’ (Australian Bureau of Statistics, 2012).

#### Health service use factors

We included the number of GP visits (MBS items listed under Broad Type of Service #101) in the 12 months prior to completing the survey in 2019 (categorised in approximate quartiles) and having private ancillary health insurance (as reported in the survey).

### Outcomes: MHTPs and treatments

Using linked MBS data, we initially identified women who had made a claim for at least one MHTP between 1 November 2019 and 31 December 2022. Among these women, we then identified those who had used at least one subsidised psychological treatment that can be claimed once an MHTP is in place. Full details on all the MBS item codes used to ascertain claims for MHTPs and psychological treatment are included in Supporting Information Table S1. We also identified whether women were prescribed any antidepressants (yes/no) during this period using linked PBS data (ATC code N06A). Antidepressants are the medication of choice in the treatment of depression and anxiety when pharmacotherapy is considered (Lampe, 2013). Anxiolytics (e.g. benzodiazepines) were not included, as these medicines are seldom used in young women except for short-term use (Lampe, 2013).

We had two outcome variables. We created a four-category variable to reflect the different combinations of receipt of an MHTP and/or antidepressant use: ‘no MHTP/no antidepressants’, ‘antidepressants only’, ‘MHTP only’ and ‘MHTP and antidepressants’.

Similarly, we created a four-category variable to reflect the different combinations of use of psychological treatment and/or antidepressants among women with an MHTP: ‘no psychological treatment/no antidepressants’, ‘antidepressants only’, ‘psychological treatment only’ and ‘psychological treatment and antidepressants’.

### Statistical analysis

First, we described the characteristics of women at study baseline (Survey 6, 2019) and also described the sociodemographic and health service use factors of women by their K10 categories of psychological distress. To assess the impact of loss to follow-up and missing data, we compared the characteristics of women included and excluded from the analysis using data from Survey 1 (2013).

Next, we used multinomial logistic regression to estimate unadjusted and adjusted relative risk ratios (RRRs) and 95% confidence intervals (CIs) for:

1. The associations between the exposures of interest (remoteness of residence, education level and perceived ability to manage with available income) and the outcome of use of different combinations of MHTP and/or antidepressants compared to no MHTP/no antidepressants. The adjusted model included the three exposure variables, and the K10 score and GP visits as covariates.
2. The associations between the exposures of interest and the outcome of use of different combinations of psychological treatment and/or antidepressants compared to no psychological treatment/no antidepressants (among women with an MHTP). The adjusted model included the three exposure variables, and the K10 score, GP visits and private health insurance as covariates. We included ancillary private health insurance in this model, as we hypothesised that women with insurance would be more likely to use psychological treatment because many private health funds would provide some level of reimbursement for additional treatment visits once the annual cap was reached.

All analyses were done using Stata 18.0 for Windows (StataCorp, 2023). We used the STROBE cohort checklist when writing our report (Von Elm et al., 2007).

## Results

**Figure 1.**
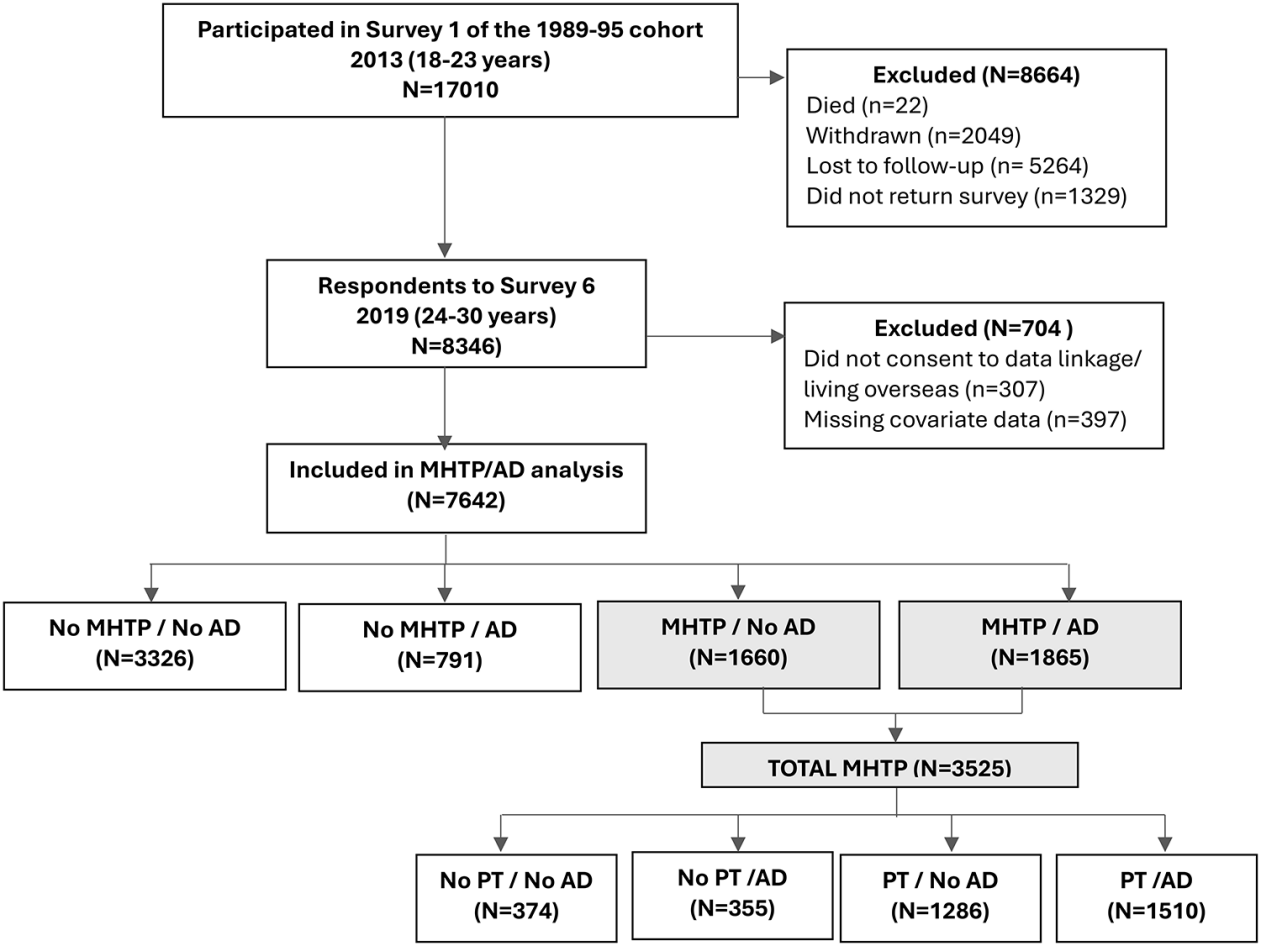
Flow chart of participants included in the study. MHTP: mental health treatment plan; AD: antidepressant; PT: psychological treatment.

Of the 17 010 women who completed Survey 1 in 2013, 8346 women also completed Survey 6 in 2019 (aged 24–29 years). In addition, we excluded women if they did not consent to data linkage or were living overseas when they completed the survey (*n* = 307) and 397 women who had missing data on one or more of the included variables, leaving 7642 women in the analysis of having an MHTP and/or antidepressants (Figure 1). Women excluded from the analysis were more likely to have lower education levels, greater difficulty managing with their available income and poorer mental health than those included (Supporting Information Table S2).

Over the study period, 1 November 2019 to 31 December 2022, 3525 (46%) women received at least one MHTP and 2656 (35%) had at least one antidepressant prescription. Just over half of the women with a prescription (*n* = 1437, 54%) were already using antidepressants prior to the study period. Of the 3525 women who had an MHTP, 2796 (79%) went on to claim at least one psychological treatment during follow-up. Fifty per cent of the study cohort had K10 scores indicating no psychological distress, 20% indicating mild levels of distress, 14% moderate levels and 15% severe levels of distress (Table 1).

**Table 1.** Descriptive characteristics at study baseline in 2019 (Survey 6, age 24–29 years) of women in the 1989–1995 cohort of the Australian Longitudinal Study on Women’s Health (*n* = 7642).

	Total population
	*N* (%)
Antidepressant use	
Yes	2656 (34.8)
No	4986 (65.2)
Mental health treatment plan (MHTP)	
Yes	3525 (46.1)
No	4117 (53.9)
Psychological treatment among women with an MHTP (*n* = 3525)	
Yes	2796 (79.3)
No	729 (20.7)
Modified Monash Model	
Metropolitan areas	5710 (74.7)
Regional centres	821 (10.7)
Large rural towns	446 (5.8)
Rural towns/remote areas	665 (8.7)
Highest qualification level	
Degree or higher	5098 (66.7)
Trade/diploma	1761 (23.0)
High school or less	783 (10.2)
Perceived ability to manage with available income	
Not too bad/easy	4522 (59.2)
Difficult some of time	2079 (27.2)
Impossible/difficult all the time	1041 (13.6)
Annual general practitioner visits	
<3 visits	1772 (23.2)
3 to 4 visits	1685 (22.0)
5 to 7 visits	1962 (25.7)
8 or more visits	2223 (29.1)
Ancillary private health insurance	
No	3545 (46.4)
Yes	4097 (53.6)
K10 score^a^	
Likely to have no psychological distress	3050 (50.4)
Likely to have mild psychological distress	1535 (20.1)
Likely to have moderate psychological distress	1075 (14.1)
Likely to have severe psychological distress	1182 (15.5)

N: number; K10 Score: Kessler Psychological Distress Scale Score.

a K10 score cut-offs for each category: no distress 10–19; mild distress 20–24; moderate distress 25–29; severe distress 30–50.

Women living in regional centres, rural towns and remote areas had similar levels of psychological distress to those living in metropolitan areas. Women who were not university-educated, reported difficulties managing on their available income, had more visits to a GP and did not have private ancillary health insurance were more likely to have moderate or severe levels of psychological distress (Supporting Information Table S3).

### MHTP and/or antidepressants

Approximately one in four women had both an MHTP and used antidepressants (*n* = 1865), 22% had an MHTP only (*n* = 1660), 10% of women used antidepressants only (*n* = 791) and 43% (*n* = 3326) did not have an MHTP or use antidepressants (Figure 1). Descriptive characteristics of the women by MHTP/antidepressant status are shown in Supporting Information Table S4. Results for the multinomial regression analyses are presented in Table 2 with women who did not receive an MHTP and did not use antidepressants as the reference group. Compared to women living in metropolitan areas, women living in a medium/small rural town or remote area were less likely to have an MHTP with or without antidepressants, and women living in large rural towns were less likely to have an MHTP only. Women living in regional centres compared to those living in metropolitan areas (adjusted relative risk ratio [aRRR] = 1.32; 95% CI = [1.02, 1.68]) were more likely to use antidepressants only.

**Table 2.** Unadjusted- and multivariable-adjusted relative risk ratios (RRR) and 95% confidence intervals (CI) for the associations between sociodemographic factors, health care use factors and the K10 Score and having a mental health treatment plan (MHTP) and/or using antidepressants in the 1989–1995 cohort of the Australian Longitudinal Study on Women’s Health (*n* = 7642).

	MODEL 1^a^ no MHTP/No AD use vs (*N* = 3326)			MODEL 2^b^ no MHTP/no AD use vs (*N* = 3326)		
	AD only (*N* = 791)	MHTP only (*N* = 1660)	MHTP and AD (*N* = 1865)	AD only (*N* = 791)	MHTP only (*N* = 1660)	MHTP and AD (*N* = 1865)
	RRR [95% CI]	RRR [95% CI]	RRR [95% CI]	RRR [95% CI]	RRR [95% CI]	RRR [95% CI]
Area of residence						
Metropolitan areas	Ref.	Ref.	Ref.	Ref.	Ref.	Ref.
Regional centres	1.22 [0.96, 1.55]	0.78 [0.64, 0.96]	1.09 [0.91, 1.31]	1.32 [1.02, 1.68]	0.82 [0.67, 1.01]	1.20 [0.98, 1.47]
Large rural towns	0.92 [0.67, 1.28]	0.58 [0.44, 0.76]	0.85 [0.67, 1.08]	0.92 [0.65, 1.29]	0.60 [0.45, 0.79]	0.87 [0.67, 1.14]
Rural towns/remote areas	0.87 [0.67, 1.15]	0.50 [0.40, 0.64]	0.79 [0.64, 0.96]	0.84 [0.63, 1.12]	0.50 [0.40, 0.64]	0.79 [0.63, 0.98]
High qualification level						
University degree or higher	Ref.	Ref.	Ref.	Ref.	Ref.	Ref.
Trade/diploma	1.84 [1.53, 2.20]	1.01 [0.87, 1.78]	1.79 [1.56, 2.04]	1.30 [1.07, 1.58]	0.91 [0.78, 1.06]	1.17 [1.01, 1.37]
High school or less	2.35 [1.86, 2.98]	1.08 [0.87, 1.33]	1.82 [1.51, 2.20]	1.58 [1.22, 2.04]	0.96 [0.77, 1.19]	1.12 [0.90, 1.38]
Perceived ability to manage with available income						
Not too bad/easy	Ref.	Ref.	Ref.	Ref.	Ref.	Ref.
Difficult some of the time	1.64 [1.37, 1.96]	1.12 [0.98, 1.29]	1.83 [1.60, 2.09]	1.06 [0.87, 1.28]	0.96 [0.83, 1.11]	1.15 [0.99, 1.33]
Impossible/difficult all the time	2.95 [2.36, 3.69]	1.65 [1.36, 2.00]	3.36 [2.83, 3.99]	1.21 [0.95, 1.56]	1.22 [0.99, 1.50]	1.29 [1.06, 1.58]
Annual general practitioner visits						
<3 visits	Ref.	Ref.	Ref.	Ref.	Ref.	Ref.
3 to 4 visits	1.70 [1.30, 2.23]	1.38 [1.17, 1.64]	1.67 [1.38, 2.03]	1.62 [1.23, 2.13]	1.34 [1.13, 1.59]	1.57 [1.28, 1.92]
5 to 7 visits	3.02 [2.35, 3.87]	1.83 [1.55, 2.16]	3.13 [2.61, 3.75]	2.82 [2.19, 3.64]	1.78 [1.50, 2.11]	2.88 [2.37, 3.48]
8 or more visits	5.26 [4.13, 6.70]	2.16 [1.82, 2.56]	6.29 [5.27, 7.50]	4.32 [3.37, 5.54]	2.00 [1.68, 2.38]	5.04 [4.18, 6.08]
K10 score^c^						
Likely to have no psychological distress	Ref.	Ref.	Ref.	Ref.	Ref.	Ref.
Likely to have mild psychological distress	2.52 [2.04, 3.11]	1.94 [1.67, 2.25]	2.51 [2.13, 2.95]	2.38 [1.92, 2.96]	1.92 [1.65, 2.23]	2.35 [1.98, 2.78]
Likely to have moderate psychological distress	4.39 [3.45, 5.57]	2.56 [2.12, 3.09]	5.99 [5.00, 7.17]	3.95 [3.09, 5.06]	2.47 [2.04, 2.99]	5.41 [4.48, 6.52]
Likely to have severe psychological distress	8.41 [6.68, 10.58]	2.34 [1.89, 2.90]	11.40 [9.49, 13.69]	6.59 [5.16, 8.42]	2.16 [1.72, 2.70]	8.98 [7.38, 10.93]

MHTP: mental health treatment plan; AD: antidepressant; N: number; RRR: relative risk ratio; CI: confidence interval; Ref: reference category; K10 score: Kessler Psychological Distress Scale-10 Score.

a Model 1 unadjusted.

b Model 2 adjusted for all variables.

c K10 score cut-offs for each category: no distress – 10–19; mild distress – 20–24; moderate distress – 25–29; severe distress – 30–50.

Women whose highest education qualification was a high school certificate were more likely to use antidepressants only than women with a university degree (aRRR = 1.58; 95% CI = [1.22, 2.04]). Difficulty managing with available income (difficult or impossible vs it being not too bad/easy) was associated with MHTP or antidepressants or both in unadjusted models; however, these associations attenuated in the adjusted models, mainly when psychological distress was added to the model. Women who had three or more annual GP visits (vs < 3 visits) were more likely to have an MHTP or antidepressants or both. Compared to women with no psychological distress, women with mild, moderate or severe levels of distress were all more likely to have an MHTP or antidepressants or both.

### Use of psychological treatment and/or antidepressants

Of the 3525 women who had an MHTP, 1510 (43%) went on to use both psychological treatment and antidepressants, 36% used psychological treatment only (*n* = 1286), 10% used antidepressants only (*n* = 355) and 11% did not use psychological treatment or antidepressants (*n* = 374) (Figure 1). Characteristics of the women by psychological treatment/antidepressant status are shown in Supporting Information Table S5. Results for the regression analyses are presented in Table 3; women who did not use psychological treatment or antidepressants were the reference group. Compared to metropolitan areas, women living in large rural towns were less likely to use psychological treatment only (aRRR = 0.56; 95% CI = [0.33, 0.92]), while those living in a medium/small rural town or remote area had a higher likelihood of antidepressant use only (aRRR = 2.00; 95% CI = [1.13, 3.53], Table 3). Women whose highest education qualification was a trade/diploma, or a high school certificate, were also more likely to use antidepressants only than women with a university degree (aRRR = 2.10; 95% CI = [1.47, 3.01] and 1.69; 95% CI = [1.06, 2.70], respectively). Associations between difficulty managing with available income and use of psychological treatment or antidepressants or both were attenuated in the adjusted models.

**Table 3.** Unadjusted and multivariable-adjusted relative risk ratios (RRR) and 95% confidence intervals (CI) for the associations between sociodemographic factors, health care use factors and the K10 Score and using psychological treatment and/or antidepressants among women who had a mental health treatment plan (November 2019 to December 2022) in the 1989–1995 cohort of the Australian Longitudinal Study on Women’s Health (*n* = 3525).

	MODEL 1^a^ no PT/no AD vs (*N* = 374)			MODEL 2^b^ no PT/no AD vs (*N* = 374)		
	AD only (*N* = 355)	PT only (*N* = 1286)	PT and AD (*N* = 1510)	AD only (*N* = 355)	PT only (*N* = 1286)	PT and AD (*N* = 1510)
	RRR [95% CI]	RRR [95% CI]	RRR [95% CI]	RRR [95% CI]	RRR [95% CI]	RRR [95% CI]
Modified Monash Model						
Metropolitan areas	Ref.	Ref.	Ref.	Ref.	Ref.	Ref.
Regional centres	1.44 [0.90, 2.32]	0.90 [0.60, 1.33]	1.25 [0.85, 1.83]	1.51 [0.93, 2.25]	0.93 [0.63, 1.39]	1.39 [0.94, 2.05]
Large rural towns	1.18 [0.66, 2.11]	0.51 [0.31, 0.85]	0.84 [0.53, 1.34]	1.12 [0.62, 2.04]	0.56 [0.33, 0.92]	0.92 [0.56, 1.49]
Rural towns/remote areas	2.19 [1.26, 3.83]	0.99 [0.60, 1.64]	1.42 [0.87, 2.30]	2.00 [1.13, 3.53]	1.04 [0.63, 1.73]	1.46 [0.89, 2.40]
Highest education qualification						
University degree or higher	Ref.	Ref.	Ref.	Ref.	Ref.	Ref.
Trade/diploma	2.78 [1.98, 3.92]	0.88 [0.66, 1.17]	1.39 [1.05, 1.84]	2.10 [1.47, 3.01]	0.92 [0.68, 1.24]	1.07 [0.79, 1.43]
High school or less	2.45 [1.57, 3.80]	0.72 [0.49, 1.06]	1.13 [0.78, 1.64]	1.69 [1.06, 2.70]	0.77 [0.52, 1.16]	0.81 [0.55, 1.20]
Perceived ability to manage with available income						
Not too bad/easy	Ref.	Ref.	Ref.	Ref.	Ref.	Ref.
Difficult some of the time	1.51 [1.07, 2.13]	0.91 [0.69, 1.19]	1.52 [1.16, 1.97]	1.03 [0.72, 1.47]	0.91 [0.69, 1.21]	1.16 [0.88, 1.54]
Impossible/difficult all the time	2.37 [1.60, 3.49]	0.79, [0.57, 1.11]	1.56 [1.13, 2.16]	1.14 [0.74, 1.76]	0.80 [0.56, 1.16]	0.89 [0.62, 1.27]
Annual general practitioner visits						
<3 visits	Ref.	Ref.	Ref.	Ref.	Ref.	Ref.
3 to 4 visits	1.47 [0.90, 2.39]	1.26 [0.90, 1.77]	1.44 [1.01, 2.04]	1.33 [0.81, 2.19]	1.20 [0.86, 1.69]	1.29 [0.90, 1.85]
5 to 7 visits	2.13 [1.36, 3.34]	1.20 [0.87, 1.65]	1.92 [1.38, 2.68]	1.86 [1.18, 2.93]	1.15 [0.83, 1.60]	1.72 [1.23, 2.41]
8 or more visits	3.39 [2.17, 5.29]	1.37 [0.99, 1.91]	3.80 [2.73, 5.29]	2.56 [1.62, 4.03]	1.34 [0.96, 1.88]	3.13 [2.23, 4.41]
Ancillary private health insurance						
No	Ref.	Ref.	Ref.	Ref.	Ref.	Ref.
Yes	0.99 [0.74, 1.33]	1.56 [1.24, 1.97]	1.34 [1.07, 1.69]	1.18 [0.87, 1.60]	1.49 [1.78, 1.89]	1.43 [1.13, 1.82]
K10 score^c^						
Likely to have no psychological distress	Ref.	Ref.	Ref.	Ref.	Ref.	Ref.
Likely to have mild psychological distress	1.32 [0.89, 1.96]	1.10 [0.83, 1.47]	1.41 [1.06, 1.89]	1.23 [0.82, 1.84]	1.13 [0.84, 1.50]	1.38 [1.02, 1.86]
Likely to have moderate psychological distress	2.44 [1.59, 3.73]	1.20 [0.85, 1.69]	2.75 [1.96, 3.85]	2.20 [1.42, 3.41]	1.24 [0.87, 1.76]	2.71 [1.92, 3.82]
Likely to have severe psychological distress	5.15 [3.32, 7.98]	1.16 [0.78, 1.72]	5.55 [3.82, 8.05]	3.97 [2.49, 6.32]	1.30 [0.86, 1.96]	5.30 [3.58, 7.86]

PT: psychological treatment; AD: antidepressant; RRR: relative risk ratio; CI: confidence interval; K10 score: Kessler Psychological Distress Scale-10 Score.

a Model 1 unadjusted.

b Model 2 adjusted for all variables that were statistically significant in the unadjusted models (area of residence, highest education qualification, perceived ability to manage with available income, ancillary private health insurance annual general practitioner visits, K10 score).

c K10 score cut-offs for each category: no distress – 10–19; mild distress – 20–24; moderate distress – 25–29; severe distress – 30–50.

Women with private health insurance were more likely to use psychological treatment only (aRRR = 1.49; 95% CI = [1.78, 1.89]) or psychological treatment and antidepressants (aRRR = 1.43, 95% CI = [1.13, 1.82]) than women without insurance; however, there was no evidence of an association between private health insurance and use of antidepressants only. In contrast, having five or more annual GP visits (vs < 3 visits) was associated with antidepressants only and psychological treatment with antidepressants.

Compared to women with no psychological distress, women with severe psychological distress were the most likely to use antidepressants only and psychological treatment with antidepressants (aRRR = 3.97; 95% CI = [2.49, 6.32] and 5.30; 95% CI = [3.58, 7.86], respectively).

## Discussion

This study investigated the use of mental health services and treatment during the COVID-19 period when levels of psychological distress were high, and governments responded by subsidising additional services. In this cohort of young women in their twenties, nearly half received at least one MHTP and one in three used antidepressants. The majority of women who had an MHTP went on to use psychological treatment. While women with the highest levels of psychological distress were the most likely to use mental health services in all combinations, we found sociodemographic disparities. Even though women living in rural and remote areas had a similar prevalence of psychological distress to women living in urban areas, women living in rural and remote areas were less likely to receive an MHTP. If they did have an MHTP, they were more likely to use antidepressants only without psychological treatment. Women with lower education levels were also more likely to use antidepressants and not have an MHTP, or if they did have an MHTP, they were more likely to use antidepressants only and not psychological treatment. Women with more annual GP visits were more likely to have an MHTP, use antidepressants or both, but if they did have an MHTP, they were more likely to use antidepressants only or psychological treatment with antidepressants (but not psychological treatment alone). Women with private health insurance were more likely to use psychological treatment (with and without antidepressants).

Geographic inequities in access to health services are well-documented in Australia, and our results support previous studies that have looked at overall access to GPs, psychiatrists and psychologists (National Rural Health Alliance, 2021; Steering Committee for the Review of Government Service Provision, 2024), use of Better Access Initiatives (Meadows et al., 2015; Pirkis et al., 2022) and prescriptions for antidepressants (Australian Bureau of Statistics, 2011). One factor likely to affect these inequities is the distribution of the mental health workforce (especially psychiatrists and psychologists) that is predominantly located in metropolitan areas or socially advantaged areas (Meadows et al., 2015; National Rural Health Alliance, 2021). In addition to issues of access to GPs and other healthcare providers who deliver psychological treatment, cost and stigma in small communities may also contribute to these geographic disparities (Cronin et al., 2025).

We found that women with lower education levels were more likely to use antidepressants if they did not have an MHTP, and if they did have an MHTP, they were more likely to use antidepressants without psychological treatment. Compared to women with higher education levels, those with less education might not have the income and/or time to access psychological treatment, particularly on an ongoing basis (Cronin et al., 2025; Cullen et al., 2023). They might also be hesitant to utilise treatments that involve ‘talking’ therapies due to lower literacy levels or perceptions of stigma, and hence may be reluctant to obtain an MHTP for this reason (Black et al., 2025; Cronin et al., 2025). It is also possible people of low socioeconomic status may receive differential treatment based on implicit bias and stereotyping by medical practitioners (especially if the consultation is short) (Job et al., 2024).

Although greater perceived difficulties managing with available income were associated with all combinations of treatment in unadjusted models, these associations attenuated in fully adjusted models. Similarly, a study using data from the Household, Income and Labour Dynamics in Australia (HILDA) Survey found no evidence of an association between financial difficulties and mental health service use by people in poor mental health (Black et al., 2025). Nevertheless, there is evidence of income constraints acting as barriers to mental health care, with one in four Australians seeking treatment by a psychologist delaying doing so because of cost (Steering Committee for the Review of Government Service Provision, 2024). An interdisciplinary review of studies investigating the bidirectional associations between poverty and mental illness highlighted the need to have policy and programme responses that integrate economic interventions with mental health care (Ridley et al., 2020).

A higher annual number of GP visits were associated with a greater likelihood of having an MHTP, using antidepressants or both, while among women who had an MHTP, having more GP visits was associated with antidepressants only or psychological treatment with antidepressants, but not psychological treatment alone. In Australia, a visit to the GP is a necessary step in receiving an MHTP, and GPs are also the major prescriber of antidepressants (Australian Institute of Health and Welfare, 2023b).

In our study, women with private health insurance were more likely to use psychological treatments (irrespective of antidepressant use). Although payments for Better Access services are subsidised by the government, private health insurers may cover some of the cost of additional visits (above the limits for Better Access treatments). In addition, motivations to take out insurance are multifaceted and include risk aversion, wanting better access to health care (e.g. greater choice of provider, shorter waiting times) and perceived current (or future) medical need (e.g. the presence of chronic conditions or multimorbidity, having children or anticipated childbirth) (Buchmueller et al., 2013). Our results concur with a Spanish study (Rocha et al., 2013) and an Australian study using data from the 45 and Up study (Dolja-Gore et al., 2018) that also found that having private health insurance increased the likelihood of visiting a psychologist. However, they differ from a study using data from the HILDA Survey that found while people with a mental health problem were less likely to have private health insurance, having insurance did not influence access to psychiatrists or psychologists (Leach et al., 2012). However, the HILDA Study presented combined results for men and women across a broad age range (15+ years), used self-report of health service use (rather than linked data) and only looked at service use among participants with a mental health problem (Leach et al., 2012).

Strengths of our study are the large sample size and the use of linked MBS and PBS data for ascertainment of the Better Access services, GP visits and antidepressant prescriptions. Limitations are that a large number of women were excluded from this analysis due to loss to follow-up, non-consent to data linkage, living overseas or missing data which may bias the analysis. However, the characteristics of the excluded women (lower education levels, greater perceived difficulties managing with available income and poorer mental health) indicate that this bias would result in an underestimate of the associations. In addition, self-report survey variables could have been measured up to 3.5 years prior to the date a woman received an MHTP during the study period. The median time from survey completion (mainly in 2019) and first MHTP was 1.9 years (interquartile range [IQR] = 1.2–2.6 years), so some of these variables (e.g. psychological distress, perceived ability managing with available income) may have changed during the intervening period.

Antidepressant use was ascertained using linked PBS data based on the date of supply of the medication; we have no information on whether the women took the medication. We also do not know if some women may have had discussions with their GP about their psychological distress that included the suggestion of an MHTP, but this was declined. Nor do we know if women who declined an MHTP differed from women who received one. We did not consider other MBS services for treatment of mental health conditions by psychiatrists, or psychologist and dietetic treatment for eating disorders. Nor do we know the extent to which women may have accessed, or been referred to, alternative mental health services or programmes (e.g. through State and Territory governments, non-government organisations, the National Disability Insurance Scheme or private mental health services).

Finally, while the cohort is broadly representative of the general population in terms of State/Territory of residence, rurality, marital status and age distribution (Loxton et al., 2018), the study population was more educated than women of the same age at the 2021 Australian Census [67% with bachelor’s degree or higher compared to 50% (Australian Bureau of Statistics, 2021)].

While it is encouraging that women with higher levels of psychological distress were more likely to receive an MHTP and receive psychological treatments (in combination with antidepressants), those living outside metropolitan areas or with lower education levels disproportionately used antidepressants without a treatment plan or psychological treatment. Further research to better understand the complex interplay of the determinants that contribute to the inequities in service delivery and take-up is needed. Evidence-based interventions to reduce these inequities in access to services should be a priority.

## Supplemental Material

sj-pdf-1-anp-10.1177_00048674251362038 – Supplemental material for Use of mental health treatment plans, psychological treatment services and antidepressants in young Australian women: A cohort study Supplemental material, sj-pdf-1-anp-10.1177_00048674251362038 for Use of mental health treatment plans, psychological treatment services and antidepressants in young Australian women: A cohort study by Louise F Wilson, Annette J Dobson, Katharine A Wallis, Jenny A Doust and Gita D Mishra in Australian & New Zealand Journal of Psychiatry
