# Supplementary material for: Use of mental health treatment plans, psychological treatment services and antidepressants in young Australian women: A cohort study
Source: Aust N Z J Psychiatry. 2025 Aug 25;59(10):906–16. doi: 10.1177/00048674251362038 (PMC12460917; doi:10.1177/00048674251362038)
Supplement: sj-pdf-1-anp-10.1177_00048674251362038 – Supplemental material for Use of mental health treatment plans, psychological treatment services and antidepressants in young Australian women: A cohort study [file sj-pdf-1-anp-10.1177_00048674251362038.pdf]

Use of mental health treatment plans, psychological treatment services and antidepressants in young Australian women – a cohort study

SUPPLEMENTARY DATA

Table S1 Medicare Benefits Schedule items that form part of the Better Access Initiative Program

Type of service	MBS item numbers
Mental Health Treatment Plan	276, 281, 282, 291, 2700, 2701, 2702, 2715, 2717, 2710, 272, 92124, 92125, 92128, 92129, 92130, 92131, 92134, 92135, 92112, 92113, 92116, 92117, 92118, 92119, 92122, 92123, 92435, 92475, 93400, 93401, 93402, 93403, 93404, 93405, 93406, 93407, 93408, 93409, 93410, 93411, 93431, 93432, 93433, 93434, 93435, 93436, 93437, 93438, 93439, 93440, 93441, 93442
Focused Psychological Strategies	General Practitioners:<br>2721, 2723, 2725, 2727, 2729, 2731, 91818, 91819, 91842, 91843, 93300, 93301, 93302, 93303, 93304, 93305, 2733, 2735, 93287, 93288<br>Other medical practitioners:<br>283, 285, 286, 287, 371, 372, 941, 942, 91820, 91844, 91821, 91845, 93306, 93307, 93308, 93309, 93310, 93311, 93291, 93292<br>Psychologists:<br>80100, 80105, 80110, 80115, 80101, 80111, 91169, 91183, 91170, 91184, 93350, 93351, 93352, 93353, 93354, 93355, 93381, 93382, 93316, 93319<br>Occupational therapists:<br>80125, 80130, 80135, 80140, 80126, 80136, 91172, 91185, 91173, 91186, 93356, 93357, 93358, 93359, 93360, 93361, 93383, 93384, 93322, 93323<br>Social workers:<br>80150, 80155, 80160, 80165, 80151, 80161, 91175, 91187, 91176, 91188, 93362, 93363, 93364, 93365, 93366, 93367, 93385, 93386, 93326, 93327,
Psychology Therapy Services	Clinical psychologists:<br>80000, 80005, 80010, 80015, 80001, 80011, 91166, 91181, 91167, 91182, 93330, 93331, 93332, 93333, 93334, 93335, 93375, 93376, 93312, 93313,

Table S2 Comparison of Survey 1 (2012, 18-23 years) characteristics of included women and women excluded due to loss to follow-up and missing data (N=17010).

	**Included women (N=7642)**	**Women excluded due to loss to follow-up (N=9368)**	
	**N(%)**	**N(%)**	**P-value**
Area of residence (n=16838)$			0.766
Major cities	5662 (74.8)	6965 (75.1)	
Inner regional	1322 (17.5)	1571 (16.9)	
Outer regional	498 (6.6)	629 (6.8)	
Rural/Remote	83 (1.1)	108 (1.2)	
Highest qualification level (n=16827)			<.0001
Degree or higher	2067 (27.3)	1767 (19.1)	
Trade/Diploma	1763 (23.3)	2652 (28.7)	
High school or less	3751 (49.5)	4827 (52.2)	
Perceived ability to manage on available income (n=16822)			<.0001
Not too bad/Easy	3180 (42.0)	3381 (36.6)	
Difficult some of the time	2721 (35.9)	3277 (35.4)	
Impossible/Always difficult	1676 (22.1)	2587 (28.0)	
Ever diagnosis of depression or anxiety (n=16844)			<.001
No	4580 (60.4)	5344 (57.7)	
Yes	3006 (39.6)	3914 (42.3)	
K10 Score (n=16838)%			<.0001
Likely to have no psychological distress	3453 (45.4)	3549 (38.3)	
Likely to have mild psychological distress	1597 (21.1)	1940 (21.0)	
Likely to have moderate psychological distress	1122 (14.8)	1450 (15.7)	
Likely to have severe psychological distress	1411 (18.6)	2316 (25.0)	

ABBREVIATIONS: N = number; K10 score = Kessler Psychological Distress Scale-10 Score

$ Area of residence at Survey 1 was measured using the Accessibility and Remoteness Index of Australia (ARIA) as the Modified Monash Model (used at Survey 6) was not developed until 2015.

% K10 score cut-offs for each category: no distress 10-19; mild distress 20-24; moderate distress 25-29; severe distress 30-50.

Table S3 Descriptive characteristics at study baseline of women by mental health status measured using the K10 Distress Scale$ (n = 7642, Survey 6, 2019); 1989-95 cohort of the Australian Longitudinal Study on Women's Health

	Likely to have no psychological distress (N=3,850)	Likely to have mild psychological distress (N=1,535)	Likely to have moderate psychological distress (N=1,075)	Likely to have severe psychological distress (N=1,182)	p-value
Modified Monash Model					
Metropolitan areas	2,852 (74.1)	1,151 (75.0)	817 (76.0)	890 (75.3)	0.20
Regional centres	439 (11.4)	144 (9.4)	120 (11.2)	118 (10.0)	
Large rural towns	235 (6.1)	89 (5.8)	49 (4.6)	73 (6.2)	
Rural towns/remote areas	324 (8.4)	151 (9.8)	89 (8.3)	101 (8.5)	
Highest qualification level					
Degree or higher	2,833 (73.6)	1,035 (67.4)	667 (62.0)	563 (47.6)	<0.001
Trade/diploma	724 (18.8)	362 (23.6)	267 (24.8)	408 (34.5)	
High school or less	293 (7.6)	138 (9.0)	141 (13.1)	211 (17.9)	
Perceived ability to manage on available income					
Not too bad/easy	2,764 (71.8)	874 (56.9)	506 (47.1)	378 (32.0)	<0.001
Difficult some of time	846 (22.0)	466 (30.4)	373 (34.7)	394 (33.3)	
Impossible/difficult all the time	240 (6.2)	195 (12.7)	196 (18.2)	410 (34.7)	
Annual general practitioner visits					
< 3 visits	1,062 (27.6)	342 (22.3)	201 (18.7)	167 (14.1)	<0.001
3 to 4 visits	904 (23.5)	344 (22.4)	232 (21.6)	205 (17.3)	
5 to 7 visits	985 (25.6)	387 (25.2)	288 (26.8)	302 (25.5)	
8 or more visits	899 (23.4)	462 (30.1)	354 (32.9)	508 (43.0)	
Ancillary private health insurance					<0.001
No	1,641 (42.6)	746 (48.6)	518 (48.2)	640 (54.1)	
Yes	2,209 (57.4)	789 (51.4)	557 (51.8)	542 (45.9)	

ABBREVIATIONS: K10 Distress Scale = Kessler Psychological Distress Scale-10 Score; N = number

$ K10 score cut-offs for each category: no distress 10-19; mild distress 20-24; moderate distress 25-29; severe distress 30-50.

Table S4 Descriptive characteristics of women by whether they received a mental health treatment plan and/or used antidepressants between 2019 and 222 in the 1989-95 cohort of the Australian Longitudinal Study on Women's Health (n=7642).

Sociodemographic, health service use and mental health factors	No MHTP/ No AD (N=3326)	AD only (N=791)	MHTP only (N=1660)	MHTP and AD (N=1865)	p-value
Modified Monash Model					<.0001
Metropolitan areas (MM1)	2414 (72.6)	571 (72.2)	1341 (80.8)	1384 (74.2)	
Regional centres (MM2)	349 (10.5)	101 (12.8)	152 (9.2)	219 (11.7)	
Large rural towns (MM3)	220 (6.6)	48 (6.1)	71 (4.3)	107 (5.7)	
Rural towns/Remote areas (MM4-MM7)	343 (10.3)	71 (9.0)	96 (5.8)	155 (8.3)	
Highest qualification level					<.0001
Degree or higher	2385 (71.7)	443 (56.0)	1179 (71.0)	1091 (58.5)	
Trade/Diploma	662 (19.9)	226 (28.6)	332 (20.0)	541 (29.0)	
High school or less	279 (8.4)	122 (15.4)	149 (9.0)	233 (12.5)	
Perceived ability to manage on available income					<.0001
Not too bad/Easy	2220 (66.7)	398 (50.3)	1020 (61.4)	884 (47.4)	
Difficult some of the time	819 (24.6)	241 (30.5)	422 (25.4)	597 (32.0)	
Impossible/Difficult all the time	287 (8.6)	152 (19.2)	218 (13.1)	384 (20.6)	
Annual General Practitioner visits					<.0001
< 3 visits	1081 (32.5)	105 (13.3)	358 (21.6)	228 (12.2)	
3 to 4 visits	852 (25.6)	141 (17.8)	391 (23.6)	301 (16.1)	
5 to 7 visits	767 (23.1)	225 (28.4)	464 (28.0)	506 (27.1)	
8 or more visits	626 (18.8)	320 (40.5)	447 (26.9)	830 (44.5)	
K10 Score$					<.0001
Likely to have no psychological distress	2243 (67.4)	263 (33.2)	809 (48.7)	535 (28.7)	
Likely to have mild psychological distress	592 (17.8)	175 (22.1)	414 (24.9)	354 (19.0)	
Likely to have moderate psychological distress	278 (8.4)	143 (18.1)	257 (15.5)	397 (21.3)	
Likely to have severe psychological distress	213 (6.4)	210 (26.5)	180 (10.8)	579 (31.0)	

ABBREVIATIONS: N = number; MHTP = mental health treatment plan; AD = antidepressants; K10 score = Kessler Psychological Distress Scale-10 Score
$ K10 score cut-offs for each category: no distress 10-19; mild distress 20-24; moderate distress 25-29; severe distress 30-50.

Table S5 Descriptive characteristics of women who had a Mental Health Treatment Plan by whether they used psychological treatment and/or used antidepressants between 2019 and 2022 in the 1989-95 cohort of the Australian Longitudinal Study on Women's Health (n=3525).

	No PT/ No AD (N = 374)	AD only (N = 355)	PT only (N = 1286)	PT and AD (N = 1510)	p-value
Modified Monash Model					<.001
Metropolitan areas (MM1)	292 (78.1)	247 (69.6)	1049 (81.6)	1137 (75.3)	
Regional centres (MM2)	36 (9.6)	44 (12.4)	116 (9.0)	175 (11.6)	
Large rural towns (MM3)	25 (6.7)	25 (7.0)	46 (3.6)	82 (5.4)	
Rural towns/Remote areas (MM4-MM7)	21 (5.6)	39 (11.0)	75 (5.8)	116 (7.7)	
Highest qualification level					<.001
Degree or higher	254 (67.9)	157 (44.2)	925 (71.9)	934 (61.9)	
Trade/Diploma	79 (21.1)	136 (38.3)	253 (19.7)	405 (26.8)	
High school or less	41 (11.0)	62 (17.5)	108 (8.4)	171 (11.3)	
Perceived ability to manage on available income					<.001
Not too bad/Easy	220 (58.8)	156 (43.9)	800 (62.2)	728 (48.2)	
Difficult some of the time	98 (26.2)	105 (29.6)	324 (25.2)	492 (32.6)	
Impossible/Difficult all of the time	56 (15.0)	94 (26.5)	162 (12.6)	290 (19.2)	
Annual General Practitioner visits					<.001
< 3 visits	93 (24.9)	44 (12.4)	265 (20.6)	184 (12.2)	
3 to 4 visits	85 (22.7)	59 (16.6)	306 (23.8)	242 (16.0)	
5 to 7 visits	105 (28.1)	106 (29.9)	359 (27.9)	400 (26.5)	
8 or more visits	91 (24.3)	146 (41.1)	356 (27.7)	684 (45.3)	
Ancillary private health insurance					<.001
No	207 (55.3)	197 (55.5)	569 (44.2)	724 (47.9)	
Yes	167 (44.7)	158 (44.5)	717 (55.8)	786 (52.1)	
K10 Score$					<.001
Likely to have no psychological distress	192 (51.3)	107 (30.1)	617 (48.0)	428 (28.3)	
Likely to have mild psychological distress	91 (24.3)	67 (18.9)	323 (25.1)	287 (19.0)	
Likely to have moderate psychological distress	53 (14.2)	72 (20.3)	204 (15.9)	325 (21.5)	
Likely to have severe psychological distress	38 (10.2)	109 (30.7)	142 (11.0)	470 (31.1)	

ABBREVIATIONS: N = number; PT = psychological treatment; AD = antidepressants; K10 score = Kessler Psychological Distress Scale-10 Score

$ K10 score cut-offs for each category: no distress 10-19; mild distress 20-24; moderate distress 25-29; severe distress 30-50.
